# Post-stroke treatment with argon attenuated brain injury, reduced brain inflammation and enhanced M2 microglia/macrophage polarization: a randomized controlled animal study

**DOI:** 10.1186/s13054-019-2493-7

**Published:** 2019-06-03

**Authors:** Jingjin Liu, Kay Nolte, Gary Brook, Lisa Liebenstund, Agnieszka Weinandy, Anke Höllig, Michael Veldeman, Antje Willuweit, Karl-Josef Langen, Rolf Rossaint, Mark Coburn

**Affiliations:** 1 Department of Anesthesiology, Medical Faculty RWTH Aachen University, Aachen, Germany; 2 Department of Neuropathology, Medical Faculty RWTH Aachen University, Aachen, Germany; 3 Department of Neurosurgery, Medical Faculty RWTH Aachen University, Aachen, Germany; 4 Institute of Neuroscience and Medicine (INM-4), Forschungszentrum Jülich, Jülich, Germany

**Keywords:** Argon, Noble gases, Neuroprotection, Infarction, middle cerebral artery

## Abstract

**Background:**

In recent years, argon has been shown to exert neuroprotective effects in an array of models. However, the mechanisms by which argon exerts its neuroprotective characteristics remain unclear. Accumulating evidence imply that argon may exert neuroprotective effects via modulating the activation and polarization of microglia/macrophages after ischemic stroke. In the present study, we analyzed the underlying neuroprotective effects of delayed argon application until 7 days after reperfusion and explored the potential mechanisms.

**Methods:**

Twenty-one male Wistar rats underwent transient middle cerebral artery occlusion or sham surgery randomly for 2 h using the endoluminal thread model. Three hours after transient middle cerebral artery occlusion induction and 1 h after reperfusion, animals received either 50% vol Argon/50% vol O_2_ or 50% vol N_2_/50% vol O_2_ for 1 h. The primary outcome was the 6-point neuroscore from 24 h to d7 after reperfusion. Histological analyses including infarct volume, survival of neurons (NeuN) at the ischemic boundary zone, white matter integrity (Luxol Fast Blue), microglia/macrophage activation (Iba1), and polarization (Iba1/Arginase1 double staining) on d7 were conducted as well. Sample size calculation was performed using nQuery Advisor + nTerim 4.0. Independent *t* test, one-way ANOVA and repeated measures ANOVA were performed, respectively, for statistical analysis (SPSS 23.0).

**Results:**

The 6-point neuroscore from 24 h to d7 after reperfusion showed that tMCAO Ar group displayed significantly improved neurological performance compared to tMCAO N_2_ group (*p* = 0.026). The relative numbers of NeuN-positive cells in the ROIs of tMCAO Ar group significantly increased compared to tMCAO N_2_ group (*p* = 0.010 for cortex and *p* = 0.011 for subcortex). Argon significantly suppressed the microglia/macrophage activation as revealed by Iba1 staining (*p* = 0.0076) and promoted the M2 microglia/macrophage polarization as revealed by Iba1/Arginase 1 double staining (*p* = 0.000095).

**Conclusions:**

Argon administration with a 3 h delay after stroke onset and 1 h after reperfusion significantly alleviated neurological deficit within the first week and preserved the neurons at the ischemic boundary zone 7 days after stroke. Moreover, argon reduced the excessive microglia/macrophage activation and promoted the switch of microglia/macrophage polarization towards the anti-inflammatory M2 phenotype. Studies making efforts to further elucidate the protective mechanisms and to benefit the translational application are of great value.

## Introduction

Every year, approximately 795,000 people experience a new or recurrent stroke, of which 87% are ischemic strokes [1]. Stroke has become one of the most frequent causes of death as well as a leading cause of major disability worldwide [2]. The thrombolytic agent tissue plasminogen activator (tPA) is the only FDA-approved drug therapy for acute ischemic stroke [3]. However, patients who are eligible for tPA treatment are only < 5% of all patients with acute ischemic stroke [4]. Moreover, tPA has several shortcomings. Notably, 2 to 7% of the patients who received tPA treatment suffer from symptomatic hemorrhagic transformation, which is a devastating complication of intravenous thrombolysis treatment and is associated with high mortality [5, 6]. Endovascular thrombectomy was shown to be a new promising treatment in patients with acute ischemic stroke. However, the indication of this therapy is so far limited to large-vessel occlusion [7]. Furthermore, access to the treatment is also limited by resources. Thus, there is an urgent need to develop novel and effective treatment strategies.

Inflammation is a vital contributor to the pathophysiology of ischemic stroke [8]. The innate immune system is activated in brain damage caused by ischemia, and inflammatory signaling is involved in all stages of stroke, from the early damaging events to the late regenerative processes underlying brain tissue repair [9]. Excessive activated native microglia and recruited macrophages in the central nervous system (CNS) induced by stroke could impair brain recovery by initiating the inflammatory cascade and releasing a considerable amount of pro-inflammatory cytokines that hinder neuronal repair [10]. For example, it was reported that the tumor necrosis factor (TNF)-α, a well-known pro-inflammatory cytokine, could regulate the downstream signaling pathways and result in activation of cellular suicide programs, including the prototype of programmed cell death, apoptosis, as well as the execution of programmed necrosis (necroptosis) [11]. An increasing amount of evidence suggest that strategies targeting microglia/macrophage activation would likely be effective in controlling injury, improving brain recovery following stroke, and thus offer the prospect of new stroke therapies [9, 10].

However, microglia also contribute to tissue repair and remodeling by clearing up debris and producing anti-inflammatory cytokines and growth factors [12], thus could be beneficial for the functional recovery after cerebral ischemia [13, 14]. With the introduction of the concept of microglia/macrophage polarization, the dual role of microglia/macrophages can be explained: The “classically activated” M1 microglia/macrophage is typically assumed to promote brain damage, whereas the “alternatively activated” M2 phenotype possesses neuroprotective properties [15, 16]. In particular, the vital roles of M2 microglia/macrophage in promoting brain restorative processes, including neurogenesis, axonal remodeling, angiogenesis, oligodendrogenesis, and remyelination [17], indicate that promoting the switch of microglia/macrophages towards M2 phenotype may serve as a new promising therapeutic approach in ischemic stroke treatment.

In recent years, the noble gas argon has demonstrated neuroprotective effects in an array of in vivo and in vitro models [18–27]. However, the mechanisms by which argon exerts its neuroprotective characteristics remain unclear and are needed to be elucidated [28]. Accumulating evidence imply that argon may exert neuroprotective effects via modulating the activation and the polarization of microglia/macrophages after ischemic stroke. In models of LPS-induced inflammation in microglia cell cultures and cortical neuronal cell cultures subjected to oxygen and glucose deprivation (OGD), argon administration suppressed the expression of pro-inflammatory cytokines, such as interleukin (IL)-1β, TNF-α, and IL-6 [24, 29]. Moreover, in models of apoptosis in human neuroblastoma cells and ischemia-reperfusion injury in retina, argon was proven to mediate neuroprotective effects via inhibiting the toll-like receptor (TLR)2/TLR4/signal transducer and activator of transcription (STAT)3/nuclear factor kappa B (NF-ҡB) pathway [25]. Interestingly, the TLRs and the NF-ҡB were verified to be essential for microglia-mediated inflammation [8, 10]. Notably, the STAT family members are considered to have crucial roles in regulating M1 or M2 gene expression and the phenotypic switching of microglia/macrophages [10, 17, 30]. However, the effects of argon on microglia/macrophage activation in the context of ischemic stroke are barely understood. Especially, the roles of microglia/macrophage polarization, in particular the roles of M2 microglia/macrophage in argon’s neuroprotective effects, have not been discussed yet.

In a previous study, Ryang and colleagues showed that argon administration for 1 h until reperfusion, with an 1 h delay after transient middle cerebral artery occlusion (tMCAO) induction, significantly reduced infarct volume and composite adverse outcome 24 h after reperfusion [18]. However, controversial results were found in other two studies [31, 32]. Against this backdrop, the following questions will be explored in the present study: (1) does argon exert neuroprotective effects when a treatment strategy which is closer to the actual clinical situation (argon administration 3 h after stroke onset and 1 h after reperfusion) is applied and when an extended observation (until 7 days after reperfusion) is performed; (2) whether argon treatment affects general microglia/macrophage activation in rat brain after ischemic stroke; (3) how is the effect of argon on M2 microglia/macrophage polarization in the context of ischemic stroke.

## Materials and methods

### Animals and treatment groups

A total of 21 male Wistar rats (body weight 290–390 g; Charles River, Sulzfeld, Germany) were housed for at least 1 week before surgery with free access to food and water on a 12-h light/dark cycle. A parallel design was applied to the present study with the allocation ratio of approximately 1:1 for the intervention groups. Animals were randomly assigned by drawing lots. In order to ensure the allocation concealment, animals were sequentially numbered, the assignments were enclosed in sealed envelopes and were not accessible to the researchers who were responsible for animal care as well as for the behavioral and histological assessments.

### tMCAO procedure

The tMCAO procedure was performed as described before [18] and was modified to fit the present experimental design. Anesthesia was induced by intraperitoneal injection of the combination of midazolame (2 mg/kg) (Ratiopharm, Ulm, Germany), medetomidine (0.15 mg/kg) (Zoetis, Florham Park, NJ, USA), and fentanyl (0.005 mg/kg) (Rotexmedica, Trittau, Germany) and maintained by hourly intraperitoneal injection of 0.03–0.05 ml of the above-mentioned anesthetics combination [18, 19]. Antagonists were not used in the anesthesia recovery period. Animals were intubated and ventilated with 50% vol N_2_/50% vol O_2_. A polyethylene catheter was inserted into tail artery to measure blood pressure and to take blood samples for blood gas analysis. Blood gas analysis was conducted for each animal at four time points (5 min after tail artery catheter insertion, after tMCAO induction, after onset of reperfusion and after the beginning of gas application) to ensure adequate ventilation. Electrocardiographic needle electrodes were placed for continuous heart rate monitoring.

Briefly, a silicone-coated 4-0 nylon monofilament was introduced via left common carotid artery into the internal carotid artery and advanced until resistance was felt, and the cerebral blood flow measurement of the left side showed a significant drop. Reperfusion was accomplished by withdrawal of the filament 2 h after tMCAO induction. One hour after reperfusion, animals received either 50% vol Argon/50% vol O_2_ (Air Liquide, Paris, France) or 50% vol N_2_/50% vol O_2_ for 1 h. During the entire surgical procedure and treatment, body temperature was maintained at 37–37.5 °C through a feedback-controlled heating pad (Physitemp Instruments, Clifton, NJ, USA). Analgesic treatment (Flunixin, 1 mg/kg s.c.) was carried out daily from the day of surgery until d3.

### Assessment of regional cerebral blood flow (CBF)

CBF assessment via the left and right middle cerebral artery was performed with a laser Doppler flowmeter (Moor Instruments, Axminster, Devon, United Kingdom) during the entire tMCAO procedure and treatment to ensure appropriate middle cerebral artery occlusion and reperfusion. The laser Doppler probes were placed on the animal’s skull approximately 1 mm posterior to the bregma and 5 mm lateral to the midline. Baseline measurement was taken 5 min after tail artery catheter insertion.

### 6-Point neuroscore

Neurological function was examined by a blinded investigator using the 6-point neuroscore as reported previously [18]. Neuroscores were assessed daily from 24 h to d7. The 6-point neuroscore was graded in six levels from 0 to 5 (5 = normal motor function, no neurologic deficit; 4 = flexion of torso and contralateral forelimb when lifted by the tail; 3 = decreased resistance to lateral push without circling; 2 = circling to the contralateral side against resistance when tugged by the tail on a flat surface; 1 = circling spontaneously to the contralateral side; 0 = no spontaneous motor activity, loss of walking or righting reflex).

### Tissue sampling

Rats were euthanized on d7. They were terminally anesthetized and brains were removed and were immediately fixed in 4% paraformaldehyde. Forty-eight hours later, rat brains were sectioned into 2-mm coronal blocks, and in total, six blocks were embedded per animal. A 2-μm section from the start of each block was collected for hematoxylin-eosin staining and the following infarct volume measurement. Sections from the fourth block (1 mm to − 1 mm in the coronal plane from Bregma) were used for other stainings (see below).

### Infarct volume measurement

Sections were stained with routine hematoxylin-eosin and were visualized and photographed with an Axiovert 200 M microscope (ZEISS, Oberkochen, Germany) with a × 10 objective. Images were analyzed using ZEN software (ZEISS, Oberkochen, Germany). The infarct volume was calculated by an indirect method of subtracting the non-lesioned volume of the ipsilateral hemisphere from the non-lesioned volume of the contralateral hemisphere and was normalized to the volume of the contralateral hemisphere of the same section.

### Luxol fast blue staining

Two-micrometer sections were stained using Luxol fast blue solution according to the protocol provided by the manufacturer (Abcam, Cambridge, UK). The sections were photographed with the EVOS FL Auto Imaging System (Life Technologies, Carlsbad, CA, USA) using a × 10 objective, and images were analyzed using ImageJ software in regions of interests (ROIs) within external capsule and striatum by measuring the positively stained area [33]. Values were normalized to the positively stained area of the contralateral hemisphere of the same section.

### Immunohistochemistry

Two-micrometer sections were cut from paraffin-embedded brain blocks and were placed on silane-coated slides. Sections were dewaxed, rehydrated, and heated in citrate buffer for antigen retrieval. Nonspecific binding was blocked by incubating sections in phosphate buffered saline containing either 1% normal goat serum or 1% BSA. The following primary antibodies were used: mouse anti-neuronal nuclear antigen (NeuN) (1:200; Merck Millipore, Billerica, MA, USA), rabbit anti-ionized calcium binding adaptor molecule 1 (Iba1) (1:500; Wako Chemicals, Neuss, Germany), and goat anti-Arginase1 (Arg1) (1:250; Santa Cruz Biotechnology, Dallas, TX, USA). Sections of NeuN immunostaining and Arg1/Iba1 double immunofluorescence were photographed with the Axioplan microscope (ZEISS, Oberkochen, Germany) at × 40 objective using the Zen software (ZEISS, Oberkochen, Germany). For NeuN immunostaining, ROIs were set to the cortex and subcortex of ischemic boundary zone (IBZ). Nuclei were counted in two images from cortex and three images from subcortex of IBZ. Values were normalized to the number of neurons counted in images collected from identical locations in the contralateral hemisphere of the same slice. For Arg1/Iba1 double immunofluorescence, three images from the inner boundary of the infarction were randomly collected, and values were presented as percentage of Arg1^+^ Iba1^+^ cells compared to Iba1^+^ cells of the same image. For Iba1 immunostaining, sections were photographed with EVOS FL Auto Imaging System (Life Technologies, Carlsbad, CA, USA) using a × 10 objective. Images were analyzed using ImageJ software by measuring the positive area of Iba1 immunostaining in the affected hemisphere. Values were normalized to the total area of the contralateral hemisphere of the same section.

### Quantification and statistical analysis

The 6-point neuroscore from 24 h to d7 after reperfusion was determined as the primary outcome. Sample size was calculated based on prior studies [34, 35]. The calculation was performed using nQuery Advisor + nTerim 4.0 (Statistical Solutions, Saugus, MA, USA). The portal “repeated measures for two means” was selected. The number of levels was set to be 7. An average difference of 0.87 between tMCAO Ar and tMCAO N_2_ groups was expected; the standard deviation was assumed to be 0.91 and the between level correlation to be 0.3. The significance was set to 5% and the statistical power to 80%. Thus, the minimum sample size for each group was defined as *n* = 7. All data were expressed as mean ± SD. Normality of the data was tested by Shapiro-Wilk test and homogeneity of variance was tested by Levene’s test. Independent *t* Test was used to perform the comparison between two groups (tMCAO Ar v.s. tMCAO N_2_ group). One-way ANOVA was applied to assess the comparison between four groups, and if necessary, Bonferroni post hoc test was used for following multiple comparisons. Courses of left and right CBF, MAP (mean arterial blood pressure) and HR (heart rate), blood gas analysis, and neuroscore were compared using repeated measures ANOVA. All calculations were performed using SPSS 23.0 (IBM, Chicago, IL). *p* < 0.05 was considered statistically significant.

## Results

**Fig. 1:**
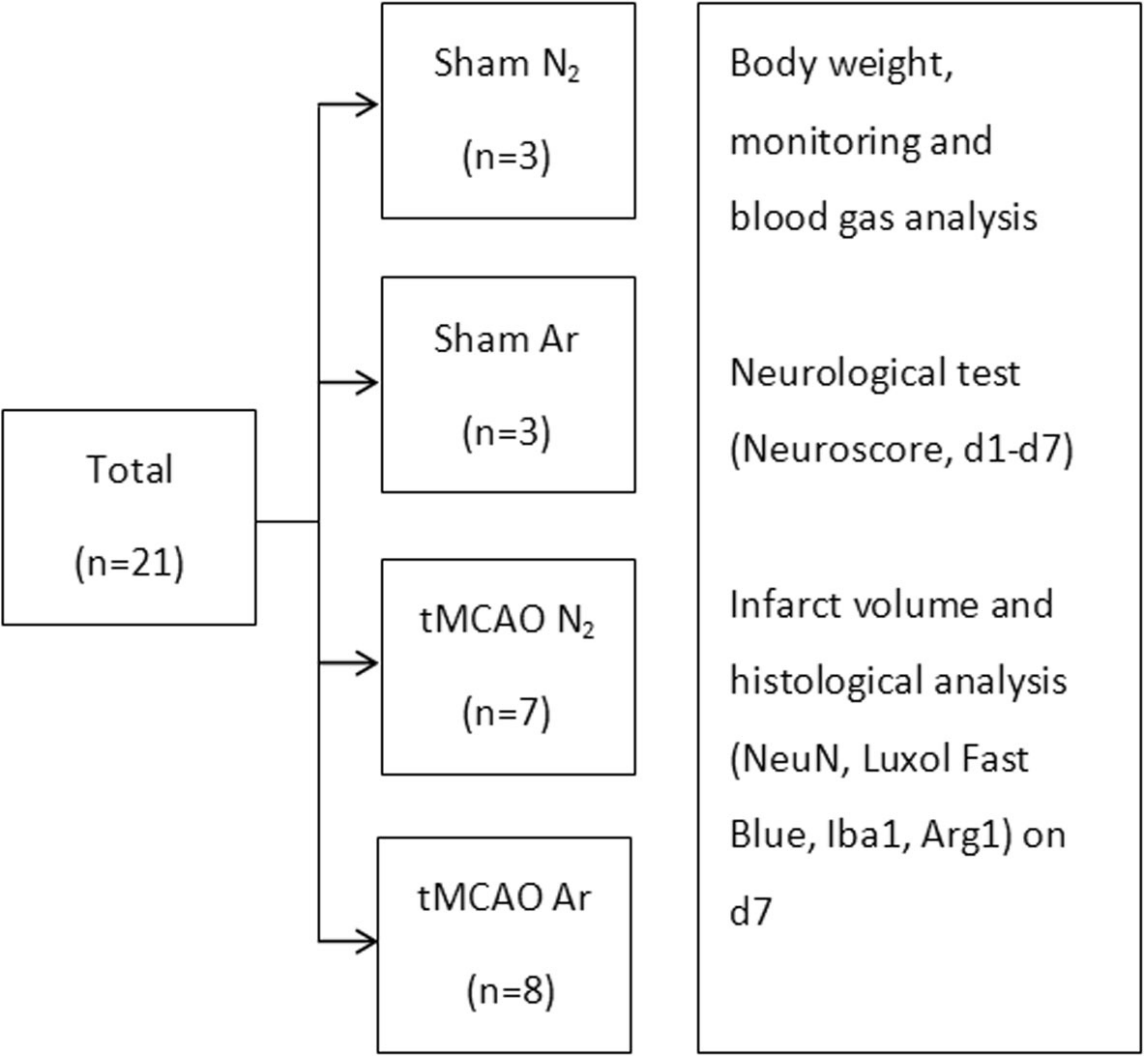
Flowchart of animal enrollment and experimental design

All animals survived until being euthanized. Thus, the data from a total of 21 animals (*n* = 8 for tMCAO Ar group, *n* = 7 for tMCAO N_2_ group, *n* = 3 for sham Ar group, and *n* = 3 for sham N_2_ group) underwent final statistical analyses. The number of animals enrolled in each group as well as the subsequent outcome measures were listed in Fig. 1.

### Monitoring, blood gas analysis, and body weight

**Fig. 2:**
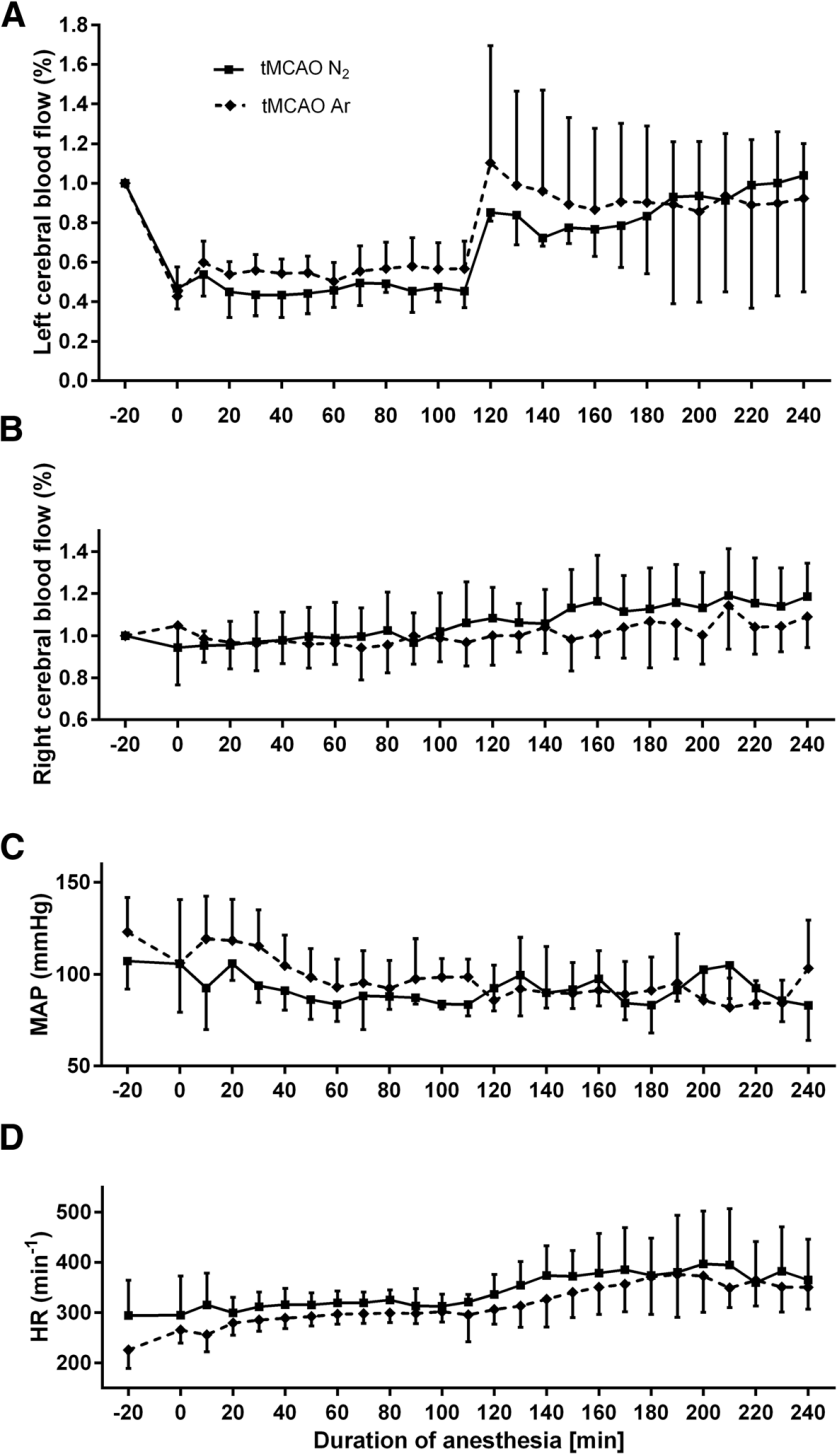
Left cerebral blood flow (CBF) (**a**) and right cerebral blood flow (**b**) as percentage of baseline value; mean arterial blood pressure (MAP) in mmHg (**c**); heart rate (HR) in beats per minute (**d**). The courses of left CBF, right CBF, MAP, and HR did not differ between tMCAO Ar and tMCAO N_2_ groups. B = baseline, 5 min after tail artery catheter insertion, time point 0 = tMCAO induction, time point 120 = start of reperfusion, time point 180 = gas application (50% vol N_2_/50% vol O_2_ for tMCAO N_2_ group, 50% vol Argon/50% vol O_2_ for tMCAO Ar group). Data were extracted in 10-min intervals. Results were represented as mean ± SD, *n* = 8 for tMCAO Ar group and *n* = 7 for tMCAO N_2_ group

The cerebral blood flow measurement of the left side showed an expected significant drop from baseline level during the tMCAO procedure. At the time point of tMCAO induction, the CBF values deceased to 42.6% ± 15.0% v.s. 46.6% ± 10.3% for tMCAO Ar and tMCAO N_2_ groups. In the reperfusion period, the values returned to baseline level. Meanwhile, the cerebral blood flow measurement of the right side showed steady values during the complete surgery procedure, which excluded the possibility of subarachnoid hemorrhage. Neither courses of left CBF, nor courses of right CBF differed between tMCAO Ar and tMCAO N_2_ groups (*p* = 0.66 for left CBF and *p* = 0.49 for right CBF) (Fig. 2a, b). Neither courses of mean arterial blood pressure (MAP) nor courses of heart rate (HR) differed between tMCAO Ar and tMCAO N_2_ groups (*p* = 0.37 for MAP and *p* = 0.14 for HR) (Fig. 2c, d). At time points before tMCAO, after tMCAO induction, after onset of reperfusion and after the beginning of gas application (50% vol N_2_/50% vol O_2_ for tMCAO N_2_ group, 50% vol Argon/50% vol O_2_ for tMCAO Ar group), none of the parameters of blood gas analysis (pH, pCO_2_, pO_2_, cK^+^, and cNa^+^) showed significant difference between tMCAO Ar and tMCAO N_2_ groups (*p* = 0.87 for pH, *p* = 0.58 for pCO_2_, *p* = 0.84 for pO_2_, *p* = 0.53 for cK^+^, and *p* = 0.81 for cNa^+^) (Table 1). The baseline body weights of animals were similar in tMCAO Ar and tMCAO N_2_ groups (350.5 ± 20.9 g v.s. 326.7 ± 33.8 g, *p* = 0.12).

**Table 1:** Blood gas analysis

–	Time 1	Time 2	Time 3	Time 4
pH				
tMCAO N_2_	7.40 (0.04)	7.44 (0.01)	7.39 (0.03)	7.41 (0.03)
tMCAO Ar	7.40 (0.06)	7.42 (0.03)	7.39 (0.01)	7.41 (0.04)
pCO_2_ (mmHg)				
tMCAO N_2_	47.7 (5.1)	41.7 (3.6)	48.7 (3.2)	45.6 (4.5)
tMCAO Ar	43.9 (7.6)	44.4 (5.4)	48.3 (5.9)	43.7 (4.7)
pO_2_ (mmHg)				
tMCAO N_2_	160.0 (17.1)	166.3 (8.1)	165.0 (17.7)	165.7 (23.4)
tMCAO Ar	162.0 (7.0)	169.3 (11.8)	164.7 (6.1)	165.3 (18.9)
cK^+^ (mmol L^−1^)				
tMCAO N_2_	4.8 (0.2)	5.0 (0.6)	5.3 (0.6)	4.9 (0.2)
tMCAO Ar	4.8 (0.5)	5.4 (0.4)	5.0 (0.1)	4.5 (0.2)
cNa^+^ (mmol L^−1^)				
tMCAO N_2_	139.7 (0.6)	145.0 (1.0)	139.0 (1.7)	143.3 (2.1)
tMCAO Ar	141.7 (2.9)	138.0 (3.0)	142.0 (1.7)	144.7 (0.6)

Results were represented as mean (SD), *n* = 8 for tMCAO Ar group and *n* = 7 for tMCAO N_2_ group. Time 1 = baseline, 5 min after tail artery catheter insertion, Time 2 = after tMCAO induction, Time 3 = after onset of reperfusion, Time 4 = after the beginning of gas application (50% vol N_2_/50% vol O_2_ for tMCAO N_2_ group, 50% vol Argon/50% vol O_2_ for tMCAO Ar group)

### Argon alleviated neurological deficit after tMCAO

**Fig. 3:**
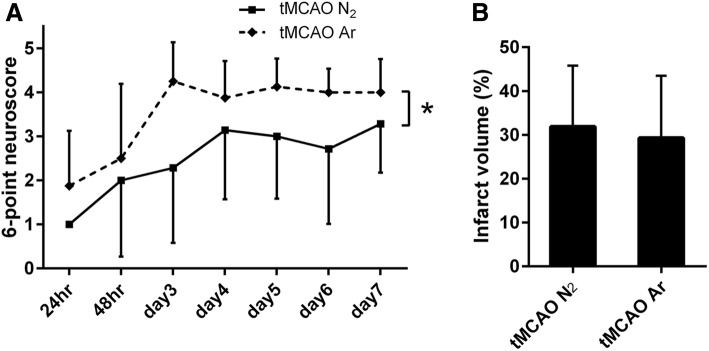
6-Point neuroscore tested daily from 24 h to d7 after reperfusion (**a**); quantification of brain infarct volume on d7 (**b**). Significance was indicated with *p < 0.05. tMCAO Ar group displayed significantly improved neurological performance compared to tMCAO N_2_ group (*p* = 0.026). Results were represented as mean ± SD, *n* = 8 for tMCAO Ar group and *n* = 7 for tMCAO N_2_ group

To assess whether argon has a functionally protective effect after ischemic stroke, we conducted the neurological function examination using a 6-point neuroscore. The 6-point neuroscore examined daily from 24 h to d7 after reperfusion showed that tMCAO Ar group displayed significantly improved neurological performance compared to tMCAO N_2_ group (*p* = 0.026) (Fig. 3a).

### Argon preserved neurons at the ischemic boundary zone after tMCAO

To detect whether argon has neuroprotective effects on rat brain tissue after stroke insult, we first analyzed the brain infarct volume 7 days after reperfusion. The border between the ischemic core and the IBZ was identified with the loss of neurons and the degradation of white matter. Quantitative analysis of the percentage of brain infarct volume did not reveal any significant difference between tMCAO Ar and tMCAO N_2_ groups (*p* = 0.73) (Fig. 3b).

**Fig. 4:**
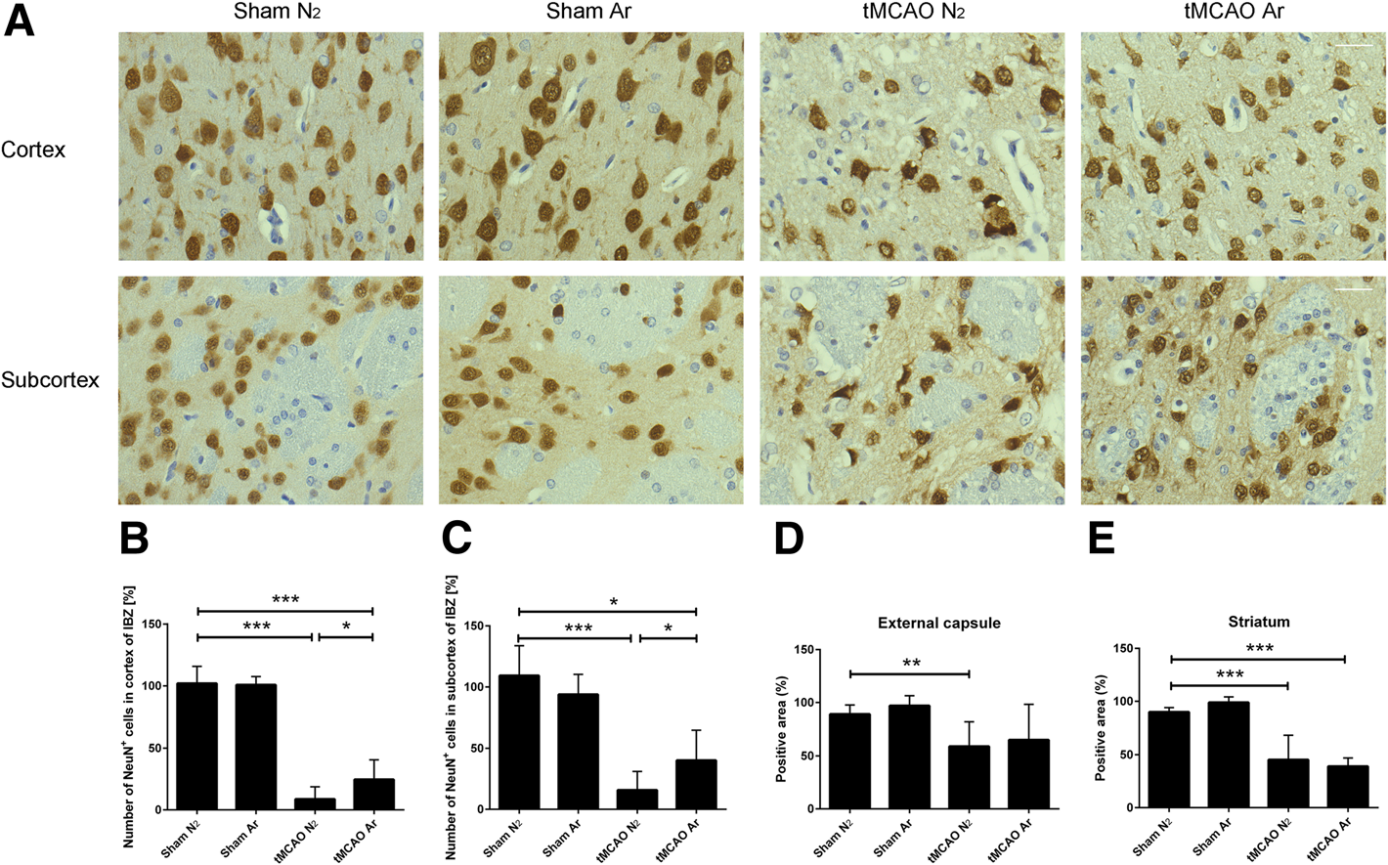
Neuronal nuclear antigen (NeuN) staining (**a**–**c**) and Luxol Fast Blue myelin staining (**d**, **e**) of rat brain samples 7 days after reperfusion. Representative images of NeuN immunohistochemistry in cortex and subcortex of ischemic boundary zone (IBZ) 7 days after reperfusion (**a**); relative number of NeuN positive cells detected in cortex (**b**) and subcortex (**c**) of IBZ; relative positive area of white matter in external capsule (**d**) and striatum (**e**). Significance was indicated with ***p < 0.001, **p < 0.01, and *p < 0.05. Argon treatment significantly increased the relative numbers of NeuN-positive cells in cortex and subcortex of IBZ (*p* = 0.010 for cortex and *p* = 0.011 for subcortex). Results were represented as mean ± SD, *n* = 8 for tMCAO Ar group, *n* = 7 for tMCAO N_2_ group, *n* = 3 for sham Ar and sham N_2_ groups

We further investigated the effect of argon on the survival of neurons at the IBZ area 7 days after reperfusion using NeuN immunostaining, which is robust and widely used to detect neurons. Quantitative analysis showed that the relative numbers of NeuN positive cells in the ROIs were significantly fewer in both tMCAO N_2_ and tMCAO Ar groups compared to Sham N_2_ group (*p* = 0.00043 for cortex and *p* = 0.00080 for subcortex of tMCAO N_2_ group, *p* = 0.00011 for cortex and *p* = 0.023 for subcortex of tMCAO Ar group). When compared to tMCAO N_2_ group, the relative numbers of NeuN-positive cells in the ROIs of tMCAO Ar group significantly increased (*p* = 0.010 for cortex and *p* = 0.011 for subcortex). (Fig. 4a–c).

Subsequently, we assessed the effect of argon on white matter damage 7 days after reperfusion using Luxol Fast Blue myelin staining. Zhou and colleagues found that the tMCAO procedure affected white matter integrity mainly within the regions of external capsule and striatum [33]. These regions were, therefore, set as ROIs in the present study. Quantitative analysis demonstrated that the relative positive area in external capsule of tMCAO N_2_ significantly decreased compared to Sham N_2_ group (*p* = 0.0025). The value of tMCAO Ar group neither differed significantly from Sham N_2_ group nor from tMCAO N_2_ group (*p* = 0.33 to Sham N_2_ group, *p* = 0.58 to tMCAO N_2_ group). In the striatum, the relative positive area of both tMCAO N_2_ and tMCAO Ar groups were significantly reduced compared to Sham N_2_ group (*p* = 0.00021 for tMCAO N_2_ group, *p* = 0.00021 for tMCAO Ar group). However, no significant difference was found between tMCAO Ar and tMCAO N_2_ groups (*p* = 0.91) (Fig. 4d, e).

### Argon suppressed microglia/macrophage activation after tMCAO

**Fig. 5:**
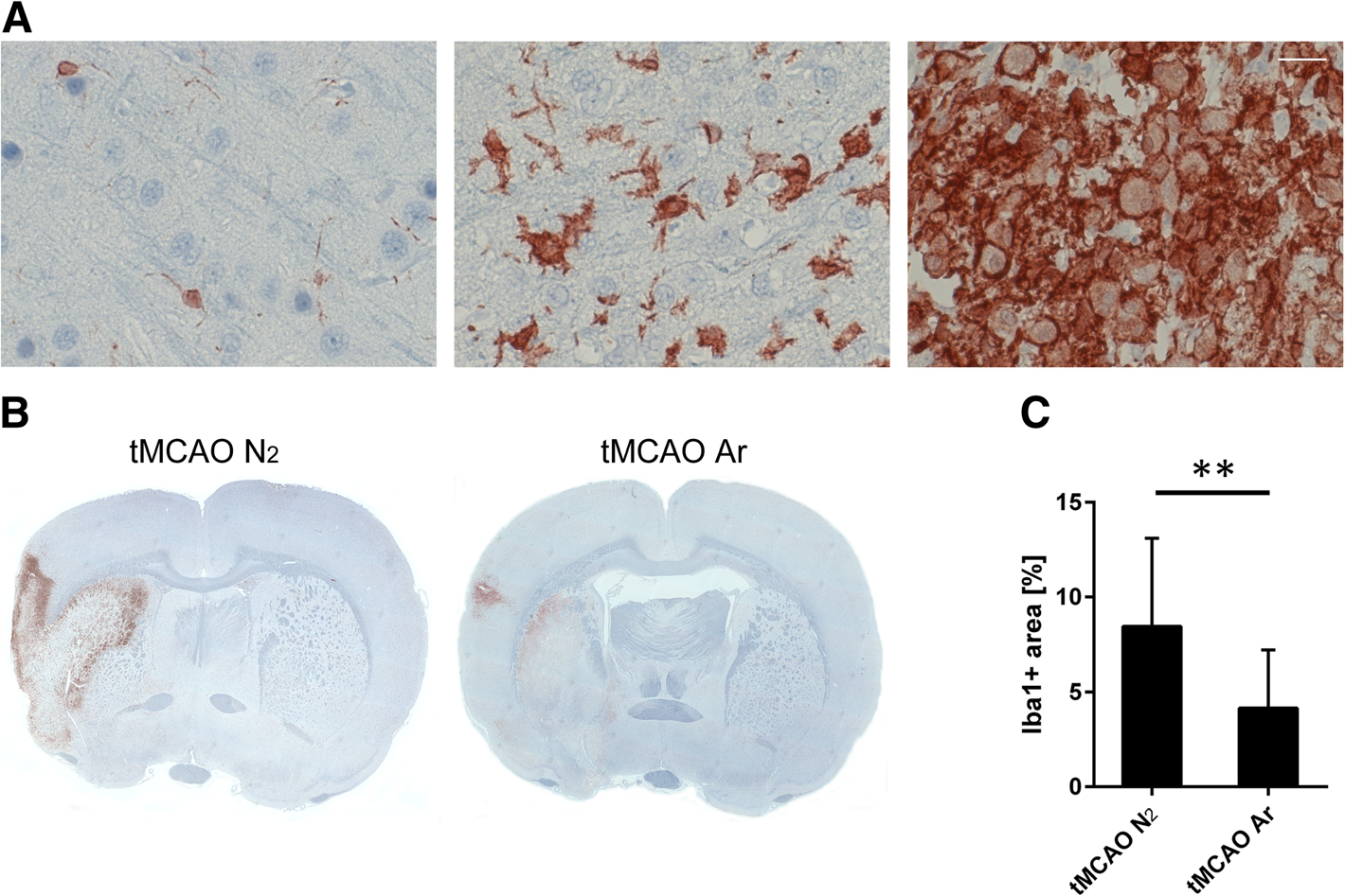
Microglia/macrophage activation with ionized calcium binding adaptor molecule 1 (Iba1) immunohistochemistry in rat brain 7 days after reperfusion. Representative images of resting microglia (**a**, left) at the contralateral cortex, intermediately activated “Stellate” microglia (**a**, middle) at the ischemic boundary zone and highly activated “amoeboid” microglia/macrophages (**a**, right) at the ischemic core; representative images of overall microglia/macrophage activation in rat brain samples (**b**); positive area of Iba1 immunostaining in the affected hemisphere (**c**). Significance was indicated with **p < 0.01. Treatment of argon significantly suppressed the general microglia/macrophage activation (*p* = 0.0076). Results were represented as mean ± SD, *n* = 8 for tMCAO Ar group and *n* = 7 for tMCAO N_2_ group

Classical resting microglia with small cell body and high degree of ramifications were observed within the non-affected areas of the brain. “Stellate” microglia, which display thicker cell body as well as intense and shortened processes with fewer ramifications and represent an intermediately activated type of microglia, were mainly found at the IBZ. Instead, the highly activated “amoeboid” microglia/macrophages, which are with features of large and round cell body as well as the loss of processes, predominated at the ischemic core (Fig. 5a). Quantitative analysis of the relative Iba1-positive area of the affected hemisphere displayed that the treatment of argon significantly suppressed general microglia/macrophage activation in rat CNS 7 days after reperfusion (*p* = 0.0076) (Fig. 5b, c).

### Argon promoted M2 microglia/macrophage polarization after tMCAO

**Fig. 6:**
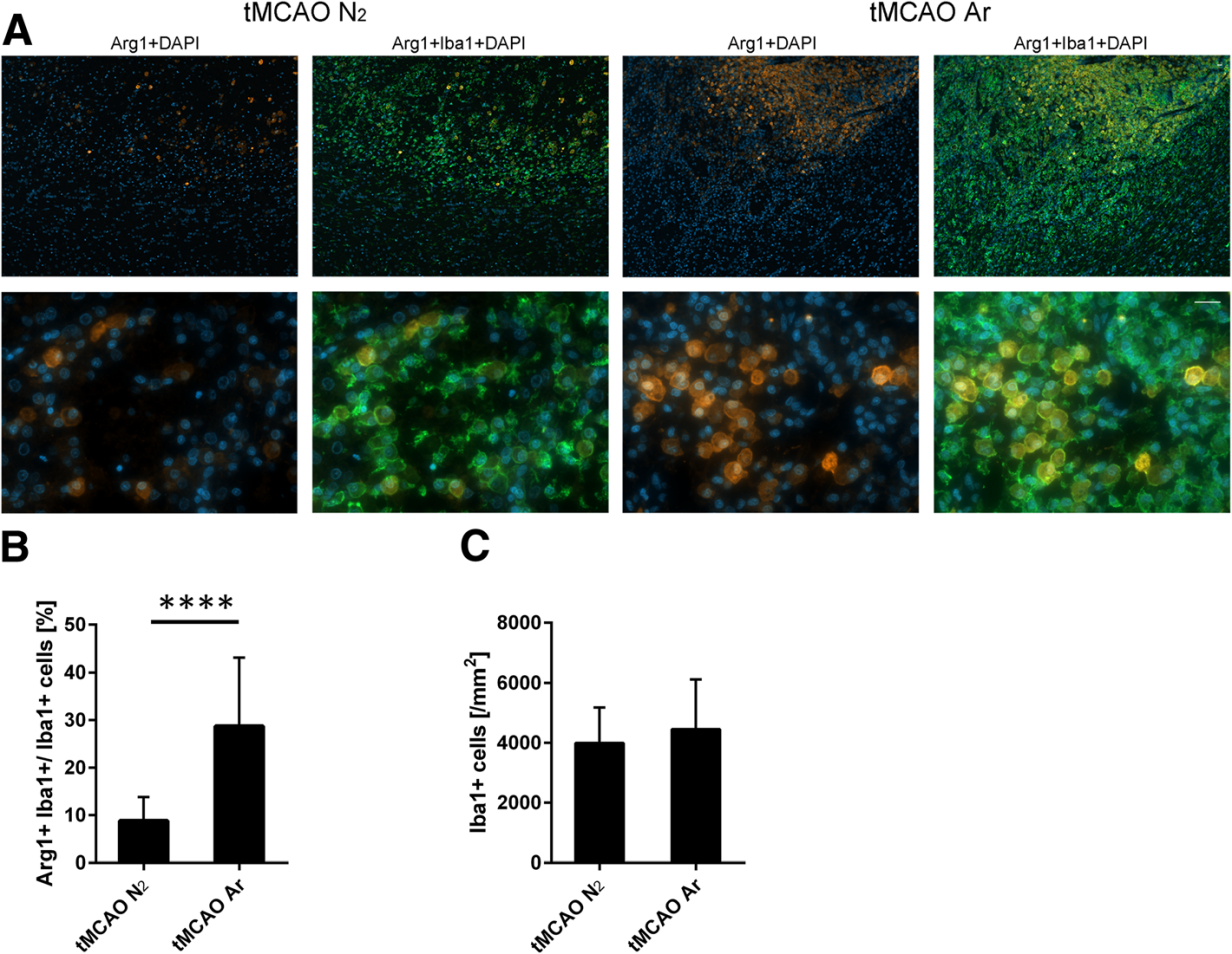
M2 microglia/macrophage polarization in rat brain 7 days after reperfusion. Rrepresentative images of Arg1 (M2 marker; orange), Iba1 (microglia marker; green), and DAPI (blue) multiple staining at the inner boundary of the infarction 7 days after reperfusion (**a**); quantification of M2 phenotype (Arg1^+^ Iba1^+^) (**b**) and microglia/macrophages (Iba1^+^) (**c**) at the inner boundary of the infarction. Significance was indicated with ****p < 0.0001. Treatment of argon significantly promoted the M2 microglia/macrophage polarization (*p* = 0.000095) in rat CNS 7 days after reperfusion. Results were represented as mean ± SD, *n* = 8 for tMCAO Ar group and *n* = 7 for tMCAO N_2_ group

Consistent with other studies [36, 37], Arg1 expression was strongest in “amoeboid” microglia/macrophages located within the ischemic core. This indicates that the strongest Arg-1 expression level is detectable in highly activated microglia/macrophages. Quantitative analysis revealed that, at the inner boundary of the infarction, the percentage of Arg1^+^Iba1^+^ cells compared to total Iba1^+^ cells of tMCAO Ar group was significantly higher compared to tMCAO N_2_ group (*p* = 0.000095) (Fig. 6b). However, the number of total Iba1^+^ cells calculated from the same images did not show any statistical difference between tMCAO Ar and tMCAO N_2_ groups (*p* = 0.30) (Fig. 6c). The results indicated that argon administration significantly promoted the M2 microglia/macrophage polarization in rat brain 7 days after reperfusion.

## Discussion

Argon treatment 3 h after stroke onset and 1 h after reperfusion improved neurological performance during the first week after ischemic stroke. The morphological analysis showed that argon administration preserved the neurons in cortex and subcortex of the ischemic boundary zone whereas the infarct volume and white matter integrity were not affected. In addition, argon alleviated the excessive microglia/macrophage activation in rat CNS. More interestingly, treatment with argon promoted the switch of microglia/macrophage polarization towards the anti-inflammatory M2 phenotype. The findings of the influences of argon on microglia/macrophage activation and polarization may lead to new understandings of the mechanisms involved in the neuroprotective effects of argon.

### The neuroprotective effects of argon after ischemic stroke

The first description of the neuroprotective properties of argon could be dated back to 1998 [38]. Thereafter, argon was proven to be an effective neuroprotective agent in a series of models, ranging from in vitro models, such as oxygen and glucose deprivation (OGD) in cortical neuronal cell cultures and brain slices, focal mechanical trauma in organotypic hippocampal slice cultures and apoptosis in human neuroblastoma cells, to in vivo models including retinal ischemia-reperfusion injury, neonatal hypoxia-ischemia brain injury, cardiac arrest, cerebral ischemia injury, and subarachnoid hemorrhage [18–27]. Within the context of cerebral ischemia injury, argon was shown to reduce the infarct volume in cortex and basal ganglia and relieve the composite adverse outcome 24 h after reperfusion when administered for 1 h until reperfusion with an 1 h delay after tMCAO induction [18]. In the present study, we further verified that, with a 3 h delay after stroke onset and 1 h after reperfusion, argon significantly alleviated neurological deficit during the first week after stroke and promoted neuronal survival in the cortex and subcortex of the ischemic boundary zone 7 days after reperfusion. However, the infarct volume and white matter integrity were not affected by argon treatment.

Although statistical analysis showed that the course of left CBF did not differ between tMCAO Ar and tMCAO N_2_ groups in the present study, the degree of CBF reduction within the infarct period and CBF recovery after reperfusion was not identical. Animals in tMCAO N_2_ group had relatively larger blood flow reduction and delayed interval to complete reperfusion. These variations between tMCAO Ar and tMCAO N_2_ groups may influence the histological and behavioral outcomes and might contribute to the inconsistency of the infarct volume and neuroscore results in the present study. Nevertheless, the calculated sample size was based on the primary outcome, namely the 6-point neuroscore from 24 h to d7 after reperfusion. Larger sample size might be needed to detect the difference in infarct volume. In humans, the size of the lesion does not always correlate with functional impairments [39]. In animal studies, the inconsistency of infarct size and functional outcomes has also been reported [40–43]. Except for the influence of the infarct core, the functional and/or structural reorganization of the remaining brain may also play important roles after ischemic stroke. In particular, the peri-infarct tissue is an important target for neurorepair and neuroprotective therapies [44]. The present findings, namely the increased number of surviving neurons at the IBZ along with the improved functional outcome generated by argon treatment, do support this point of view. Other functional and/or structural alterations including cerebral vasoconstriction, blood-brain barrier permeability, synaptic plasticity, axonal remodeling, neurogenesis, microglia/macrophage activation, astrocyte reactivity, and angiogenesis at the IBZ, even within the non-affected hemisphere, are of importance to uncover the panorama of argon’s neuroprotective effects after ischemic stroke [8, 37, 42, 45]. Meanwhile, a battery of functional assessments with respect to different aspects of the sensorimotor function as well as the cognitive function would also be beneficial to further comprehend the protective capacity of the treatment.

### The influences of argon on microglia/macrophage activation and polarization after ischemic stroke

Previous studies found that both the general microglia/macrophages and the M2 phenotype peaked at d7 after ischemic stroke insult [36, 46]. Thus, this observational time point was used in the present study to achieve the optimal exploration into the changes of microglia/macrophage activation and polarization after argon treatment.

In 2012, Fahlenkamp and colleagues first discussed the influence of argon on microglia activation using an in vitro model of LPS-induced inflammation in microglia cell cultures [29]. The authors demonstrated that administration of argon suppressed the expression of the pro-inflammatory cytokine IL-1β. Later on, using cortical neuronal cell cultures subjected to OGD, Zhang and colleagues showed that argon exposure attenuated neuronal cell death and reduced the expression of the pro-inflammatory cytokines TNF-α and IL-6 [24]. In the present study, using an in vivo cerebral ischemia injury model, we demonstrated that argon significantly attenuated the excessive microglia/macrophage activation in rat CNS caused by the insult.

In recent years, researchers have put great effort to explore the mechanisms of argon’s neuroprotective effects. So far, they are mainly related to the anti-apoptotic and the anti-oxidative stress abilities. However, the anti-neuroinflammatory potential of argon has been emerging with time [24, 28, 47]. Several receptors, such as toll-like receptors, purinergic receptors, chemokine receptor CCR2, Fc receptors, receptor for advanced glycation end products (RAGE), cysteinyl leukotriene receptor 2, galectin-3, and CD36, were shown to be involved in mediating microglia activation and its functions including inflammation, motility, migration, phagocytosis, and survival during ischemic stroke [8]. Among these receptors, the TLRs have been widely studied and were confirmed to be essential for microglia-mediated inflammation [8, 10]. In a clinical trial, Brea and colleagues found that TLR2 and TLR4 were associated with poor outcome and correlated with higher serum levels of IL-1β, TNF-α, and IL-6 in patients [48]. In animal studies, stimulation or inhibition of TLR2 and TLR4 resulted in corresponding changes in pro-inflammatory cytokines, brain infarct volume, and functional outcomes [8]. Notably, in models of apoptosis in human neuroblastoma cells and ischemia-reperfusion injury in retina, argon was proven to mediate neuroprotection via inhibiting TLR2 and TLR4 [25, 49]. Whether argon affects the microglia/macrophage activation triggered by ischemic stroke via modulating TLRs and other above-mentioned receptors and how this relates to the neuroprotective effects of the agent require further elucidation.

Over the past few years, increasing evidence manifested that M1-M2 microglia/macrophage polarization is involved in ischemic stroke insult [17, 36, 50]. Generally, the microglia/macrophages could be divided into two phenotypes, the pro-inflammatory M1 phenotype and the anti-inflammatory M2 phenotype. The M1 phenotype is characterized by a high production of pro-inflammatory cytokines including IL-1β, IL-6, TNF-α, CC-chemokine ligand (CCL) 2, and C-X-C motif chemokine (CXCL) 10, reactive oxygen species (ROS), nitric oxide (NO), and inducible nitric oxide synthase (iNOS) as well as proteolytic enzymes matrix metalloproteinase (MMP)-9 and MMP-3 [8, 10]. In contrast, the M2 phenotype is characterized by enhanced expression of Arg1, Ym1, insulin-like growth factor (IGF)-1, CD206, chitinase 3-like 3, and Fizz1 and is capable of producing anti-inflammatory cytokines, such as IL-10, transforming growth factor (TGF)-β, IL-4, and IL-13 [8, 10]. Hu and colleagues reported that local microglia and newly recruited macrophages assumed the M2 phenotype at early stages of ischemic stroke but gradually transformed into the M1 phenotype in the peri-infarct region [50]. In the present study, we first revealed that administration of argon significantly promoted the switch of microglia/macrophage polarization towards M2 phenotype after ischemic stroke. In addition, except for serving as a prominent M2 marker, Arg1 is generally considered as a protective and regeneration promoting enzyme, since it counteracts the excessive production of cytotoxic NO and is responsible for the generation of polyamines and collagen [37, 51].

A series of intracellular molecules including signal transducer and activator of transcription (STAT) family members, peroxisome proliferator-activated receptor γ (PPARγ), interferon regulatory factors (IRFs), and microRNAs were identified as key modulators of microglia/macrophage polarization [17]. Interestingly, using a model of apoptosis in human neuroblastoma cells, Ulbrich and colleagues observed that argon affected the phosphorylation and binding activity of STAT3, and that inhibition of STAT3 attenuated argon’s anti-apoptotic effect [25]. It is reasonable to assume that argon influences the activation and function of STAT3, which in turn produces an effect on microglia/macrophage polarization, and ultimately leads to behavioral and histological improvements after ischemic stroke. However, further studies will be needed to confirm this hypothesis. Moreover, some signaling pathways, such as mammalian target of rapamycin complex 1 (mTORC1) pathway and 5′ AMP-activated protein kinase (AMPK) pathway, were also identified recently to be essential for microglia/macrophage polarization after stroke [52, 53]. The roles of these molecules and pathways in the regulation of microglia/macrophage polarization and the neuroprotection induced by argon treatment should be further investigated.

### Limitations and prospective

The design of a 3 h interval of argon administration after stroke onset and the prolonged observational period would provide valuable data to guide the development of new therapeutic strategies. Furthermore, the novel findings with regard to the microglia/macrophage activation and polarization would give rise to more comprehensive understandings of the mechanisms of argon’s neuroprotective effects. However, several limitations exist in the present study. Further studies are advisable to confirm and extend the findings here.

The present study observed the effects of argon treatment up to 7 days after stroke event. According to the Stroke Therapy Academic Industry Roundtable recommendations, studies conducted at least 2 to 3 weeks or longer after stroke onset would be needed to demonstrate a sustained benefit by candidate neuroprotective agents [54]. Meanwhile, behavioral assessments with respect to different aspects of the sensorimotor function and the cognitive function, as well as evaluations of multi-faceted histological changes after treatment are of importance.

In most of the animal studies, cerebral ischemia was induced in young healthy animals. In contrast, stroke occurs in humans as a result of the natural progression of underlying diseases or risk factors, such as aging, hypertension, and diabetes [54]. Thus, studies performed in age-related or disease-related models may be of great interest and would extend the understanding of argon’s neuroprotective effect after stroke.

As mentioned above, the degree of CBF reduction within the infarct period and CBF recovery after reperfusion was not strictly the same in tMCAO Ar and tMCAO N_2_ groups. The variations may have impact on the research findings. Therefore, further studies should strive to maintain the stability and consistency of the tMCAO procedure. A larger sample size may also be beneficial.

In the present study, due to limited resources, Luxol Fast Blue myelin staining was used to assess the white matter damage after stroke. Although Luxol Fast Blue staining was proven to be an effective method to detect the demyelination in CNS [33], more specific and sensitive method such as immunohistochemical staining of myelin basic protein (MBP) may provide more valuable information.

Despite the comparability of blood gas analysis parameters between tMCAO Ar and tMCAO N_2_ groups, mild hypercapnia was detected in both groups in the present study. Hypercapnia may lead to cerebral vasodilation, increased intracranial pressure as well as cerebral edema. These effects could be even more severe when an intracranial disease, such as stroke, exists. Studies in the future should try to avoid hypercapnia so that its’ potential interference with cerebral pathophysiologic changes in the context of ischemic stroke could be excluded.

The generation of a dose-response curve is critical for neuroprotective drug candidates. 50% vol argon was used in the present study based on previous reports of in vitro and in vivo investigations [18–20]. However, there is a need to further assess the ideal concentration, timing, and duration of argon application in in vivo tMCAO models.

## Conclusions

In the present study, it was demonstrated that argon administration with a 3 h delay after stroke onset and 1 h after reperfusion significantly alleviated neurological deficit within the first week and preserved the neurons at the ischemic boundary zone 7 days after stroke. Moreover, argon reduced the excessive microglia/macrophage activation in rat CNS and promoted the switch of microglia/macrophage polarization towards the anti-inflammatory M2 phenotype. Studies making efforts to further elucidate the protective mechanisms and to benefit the translational application are of great value. Given that argon has no record of toxicity [24, 27, 28] and could exert neuroprotection via multiple mechanisms which may lead to better therapeutic effects, it is a promising agent in the future for treating ischemic stroke patients.

## Abbreviations

AMPK: 5′ AMP-activated protein kinase
Arg1: Arginase1
CBF: Cerebral blood flow
CCL: CC-chemokine ligand
CNS: Central nervous system
CXCL: C-X-C motif chemokine
HR: Heart rate
Iba1: Ionized calcium binding adaptor molecule 1
IBZ: Ischemic boundary zone
IGF: Insulin-like growth factor
IL: Interleukin
iNOS: Inducible nitric oxide synthase
IRFs: Interferon regulatory factors
MAP: Mean arterial blood pressure
MBP: Myelin basic protein
MMP: Matrix metalloproteinase
mTORC1: Mammalian target of rapamycin complex 1
NeuN: Neuronal nuclear antigen
NF-ҡB: Nuclear factor kappa B
NO: Nitric oxide
OGD: Oxygen and glucose deprivation
PPARγ: Peroxisome proliferator-activated receptor γ
RAGE: Receptor for advanced glycation end products
ROIs: Regions of interests
ROS: Reactive oxygen species
STAT: Signal transducer and activator of transcription
TGF: Transforming growth factor
TLR: Toll-like receptor
tMCAO: Transient middle cerebral artery occlusion
TNF: Tumor necrosis factor
tPA: Tissue plasminogen activator
